# Comparing the effects of highly aspherical lenslets versus defocus incorporated multiple segment spectacle lenses on myopia control

**DOI:** 10.1038/s41598-023-30157-2

**Published:** 2023-02-21

**Authors:** Hui Guo, Xianfang Li, Xiaoxiao Zhang, Haizhao Wang, Jianhua Li

**Affiliations:** Optometry and Strabismus Department, Guangzhou Aier Eye Hospital, Jinan University, 191 Huanshi Middle Road, Yuexiu District, Guangzhou, 51000 China

**Keywords:** Outcomes research, Epidemiology

## Abstract

To compare spectacle lenses with highly aspherical lenslets (HAL) versus defocus incorporated multiple segments (DIMS) on myopia progression control in 1 year. This retrospective cohort study involved data from children prescribed HAL or DIMS spectacle lenses in Guangzhou Aier Eye Hospital, China. To address the discrepancy that some children followed up at less than or more than 1 year, the standardized 1-year spherical equivalent refraction (SER) and axial length (AL) changes from baseline were calculated. The mean differences in the changes between the two groups were compared with linear multivariate regression models. Age, sex, baseline SER/AL, and treatment were included in the models. A total of 257 children who qualified for the inclusion criteria were included for the analyses (193 in the HAL group and 64 in the DIMS group). After controlling baseline variates, the adjusted mean (standard error, SE) of the standardized 1-year changes in SER for HAL and DIMS spectacle lens users were − 0.34 (0.04) D and − 0.63 (0.07) D, respectively. HAL spectacle lenses reduced myopia progression by 0.29 D (95% confidence interval [CI] 0.13 to 0.44 D) at 1 year compared to DIMS lenses. Accordingly, the adjusted mean (SE) ALs increased by 0.17 (0.02) and 0.28 (0.04) mm for children wearing HAL lenses and DIMS lenses, respectively. HAL users had 0.11 mm less AL elongation (95% CI − 0.20 to − 0.02 mm) than DIMS users. Age at baseline was significantly associated with AL elongation. Chinese children wearing spectacle lenses designed with HAL had less myopia progression and axial elongation than those wearing spectacle lenses designed with DIMS.

## Introduction

Myopia prevalence has been increasing dramatically in recent decades worldwide, particularly in East Asian countries, such as China^1^. By 2020, the prevalence of myopia among teenagers reached approximately 70% in urban cities in China^2,3^. It is estimated that by 2050, 50% of the population will have myopia, and 10% will have high myopia worldwide^4^. In particular, the predicted prevalence of myopia in China is even higher, with 84% of Chinese people having myopia^5^.

Multiple interventions have been shown to significantly slow myopia progression, including the use of some specifically designed spectacle lenses, orthokeratology lenses, multifocal soft contact lenses, etc^6^. With easy adaptation features, no ocular infection concern, and lower cost compared to contact lenses, spectacles are the most common choice for myopia control^7,8^.

Several types of spectacles have been developed to slow myopia progression^6,9,10^. Among specifically designed lenses, recent research has shed light on two types of anti-myopic spectacle lenses^11^. In 2019, the effect on myopia control with defocus incorporated multiple segments (DIMS) spectacle lenses was published. The DIMS group delayed myopia progression by 52% compared to single-vision lenses in 2 years^12^. DIMS spectacle lenses have a 9-mm central single-vision zone surrounded by a honeycomb area consisting of multiple 1.03 mm width, + 3.5 D myopic defocus segments. Later, another 2-year clinical trial found that children wearing spectacle lenses comprising rings with highly aspherical lenslets (HAL) reduced myopia progression by 55% compared with those wearing single-vision lenses^13^. Outside the central normal refractive correction zone, the HAL lenses consist of 11 rings of continuous lenslets. The lenslet produces a volume of myopic defocus image with 0.7 mm depth and approximately 1.2 mm before the retina^14^.

The two types of lenses both produce myopic defocus in the peripheral retina while maintaining a clear central vision. The two separate studies showed comparable effects of spectacles lenses with HAL or DIMS for preventing myopia progression. Since there are notable differences in the design of the lenses, we aimed to compare the myopia control efficacy between these two types of lenses.

## Methods

This was a retrospective cohort study. The ethics committee of Guangzhou Aier Eye Hospital approved the study protocol, including the waiver of informed consent (GZAIER2022IRB11). The study was performed while observing the tenants of the Declaration of Helsinki. Data were collected from the Guangzhou Aier Eye Hospital database between September 2020 and March 2022. The children were selected if they were prescribed either HAL or DIMS, were younger than 16 years, and were without strabismus, amblyopia, or other ocular or systematic abnormalities. In addition, only those who had a refraction test between 6 and 18 months after the baseline visit without changing the type of spectacles were included in the analyses.

Cycloplegic autorefraction using Topcon KR 800 (Japan) followed by subjective refraction was performed at baseline. Noncycloplegic auto- and subjective refraction were examined during the follow-up visit. If the results were ambiguous, cycloplegic refraction was conducted. Cycloplegia was induced in each eye with three drops of 0.5% tropicamide administered 5 min apart. Refraction tests were performed 15 min after the last drop. The results from subjective refraction were used for the analyses. The axial length (AL) was measured at least three times with a ZEISS IOLMaster 500 (Germany), and the average was calculated.

The primary outcome was the spherical equivalent refraction (SER) change from baseline. SER was calculated with spherical power plus half cylinder power. The secondary outcomes included the AL, spherical power, and cylinder power differences. The follow-up records within 12 ± 6 months were used to standardize the changes from baseline to 1 year according to the following calculation: changes in outcome values × (12/actual months between baseline and follow-up).

Only the results of the right eyes were reported as significant correlations were observed between the two eyes for the changes in SER (r = 0.58, *P* < 0.001), AL (r = 0.78, *P* < 0.001), spherical power (r = 0.75, *P* < 0.001), and cylinder power (r = 0.30, *P* < 0.001). To compare the baseline characteristics between the two groups, unpaired t-tests were used for continuous variables, and the chi-square test was used for the categorical variable. The differences in the outcomes between the two groups were analyzed with unpaired t-tests and linear multivariate regression models. The models included age, sex, baseline refractive values or AL, and treatment as covariates. Age, refractive values, and AL were treated as continuous variables, while sex and treatment were treated as categorical variables in the models. The linear regression models with the same covariates were repeated for the observations without any missing values as sensitivity analyses. The analyses were performed with SAS 9.4 software. Results with a two-sided *P* value ≤ 0.05 were considered statistically significant.

## Results

**Figure 1:**
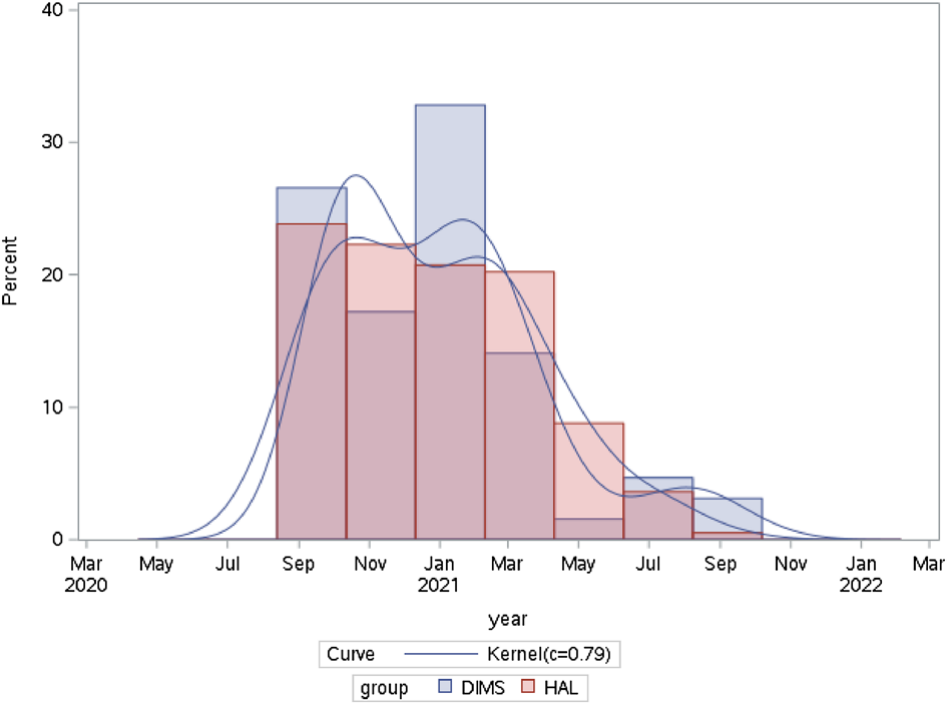
Distributions of baseline prescription dates in the two groups. *DIMS* defocus incorporated multiple segments, *HAL* highly aspherical lenslets.

A total of 403 children were prescribed HAL (n = 296) or DIMS (n = 107) lenses within the study period. There were 193 of 296 (65%) children in the HAL group and 64 of 107 (60%) children in the DIMS group who met the inclusion criteria for the final analyses. There were missing values of axial length (AL) for some observations, leaving 102 in the HAL group and 33 in the DIMS group with complete data for the sensitivity analyses. The distributions of baseline prescription dates were comparable between the two groups. The peaks of starting dates were in October 2020 and January to February 2021 (Fig. 1).

Children prescribed HAL lenses were younger than those offered DIMS lenses, with a mean (standard error, SE) age of 9.57 (0.16) years versus 10.29 (0.26) years (mean difference, − 0.72 years; 95% confidence interval, [CI], − 1.34 to − 0.09 years; *P* = 0.03). Sex, baseline refractive values, and AL were comparable between the two groups (Table 1). The mean (SE) follow-ups were 274.2 (5.5) days and 267.3 (9.3) days for the HAL and DIMS groups,respectively, and the difference was not statistically significant (mean difference, 6.9 days; 95% CI − 14.7 to 28.5 days; *P* = 0.53). Twenty-one (11%) children in the HAL group and 7 (11%) children in the DIMS group were prescribed the cycloplegic refraction at the follow-up visit.

**Table 1:** Baseline characteristics of the children in the two groups.

	HAL (n = 193)	DIMS (n = 64)	*P* value
Age, years; mean (SE)	9.57 (0.16)	10.29 (0.26)	0.03
Sex, female; n (%)	80 (41%)	28 (44%)	0.75
Spherical, diopters; mean (SE)	− 1.91 (0.09)	− 2.14 (0.18)	0.24
Cylinder, diopters; mean (SE)	− 0.65 (0.05)	− 0.61 (0.07)	0.71
SER, diopters; mean (SE)	− 2.24 (0.10)	− 2.45 (0.19)	0.32
Axial length, mm; mean (SE)	24.35 (0.09) n = 117	24.54 (0.13) n = 41	0.28

*DIMS* defocus incorporated multiple segments, *HAL* highly aspherical lenslets, *SE* standard error, *SER* spherical equivalent refraction.

The unadjusted, standardized, 1-year mean (SE) change in SER was − 0.34 (0.04) D for the HAL group and − 0.63 (0.08) D for the DIMS group. The 95% CI of the mean difference (0.29 D) between the two groups was 0.13 to 0.44 D (*P* < 0.001). After controlling for age, sex, and baseline SER, the adjusted SER change difference showed a similar result (Table 2). Accordingly, the adjusted mean (SE) difference in the spherical power change was 0.32 (0.08) D (95% CI 0.17 to 0.47 D; *P* < 0.001). In contrast, the changes in cylinder power were not significantly different between the two groups (95% CI − 0.15 to 0.06 D; *P* = 0.42). The unadjusted, standardized, 1-year mean (SE) axial elongations for children using the HAL lenses and DIMS lenses were 0.17 (0.02) mm and 0.27 (0.04) mm, respectively. The HAL group exhibited slower AL increase than the DIMS group, with a mean difference of − 0.10 mm (95% CI − 0.19 to − 0.01 mm; *P* = 0.03). The adjusted mean difference in AL change was − 0.11 mm (95% CI − 0.20 to − 0.02 mm; *P* = 0.02).

**Table 2:** Unadjusted and adjusted, standardized 1-year changes in SER and AL.

	Unadjusted				Adjusted			
	HAL, mean (SE)	DIMS, mean (SE)	95% CI	*P* value	HAL, mean (SE)	DIMS, mean (SE)	95% CI	*P* value
SER, diopters (n = 193 in the HAL group and n = 64 in the DIMS group)	− 0.34 (0.04)	− 0.63 (0.08)	0.29 (0.13 to 0.44)	< 0.001	− 0.34 (0.04)	− 0.63 (0.07)	0.29 (0.13 to 0.44)	< 0.001
AL, mm (n = 102 in the HAL group and n = 33 in the DIMS group)	0.17 (0.02)	0.27 (0.04)	− 0.10 (− 0.19 to − 0.01)	0.03	0.17 (0.02)	0.28 (0.04)	− 0.11 (− 0.20 to − 0.02)	0.02

*AL* axial length, *CI* confidence interval, *DIMS* defocus incorporated multiple segments, *HAL* highly aspherical lenslets, *SE* standard error, *SER* spherical equivalent refraction.

In the sensitivity tests without missing records for SER or AL, the adjusted, standardized, 1-year mean (SE) changes in SER were − 0.31 (0.05) D and − 0.59 (0.09) D in the HAL (n = 102) and DIMS (n = 33) groups, respectively. The mean (SE) difference between the two groups was 0.27 (0.10) D (95% CI 0.08 to 0.47 D; *P* = 0.008). The adjusted, standardized, 1-year mean (SE) ALs increased by 0.17 (0.02) mm and 0.28 (0.04) mm for children wearing HAL and DIMS lenses, respectively. DIMS users had significantly greater AL increase with a mean (SE) difference of − 0.11 (0.05) mm (95% CI − 0.20 to 0.02 mm; *P* = 0.02).

In the linear multivariate regression model, baseline age was negatively associated with axial lengthening. The standardized AL showed a 0.02 mm less increase with 1 year older at baseline (*P* = 0.03). In contrast, age was not significantly associated with SER change. Sex and baseline SER or AL values were not associated with SER or AL changes during the study period.

## Discussion

In this study, children using highly aspherical lenslets (HAL)-designed spectacle lenses reduced 0.29 D myopia progression and 0.11 mm axial length (AL) elongation compared to those wearing DIMS lenses over a standardized 1-year period after adjusting for age, sex, and baseline spherical equivalent refraction (SER) or AL. Significant differences in SER and AL changes were also detected in sensitivity tests including records without missing outcome values.

Hyperopic defocus images captured by retinal neurons can trigger eye growth, while myopic defocus inhibits eye enlargement^15,16^. Although the density of neurons in the central retina is the greatest, the accumulated number of neurons in the peripheral retina is larger than that in the fovea^16^. The area of the functional zone that creates peripheral myopic defocus is larger in the HAL lens than in the DIMS lens, which might lead to better myopia control. In addition, peripheral myopic defocus power was negatively associated with axial growth, which has been confirmed by previous studies on spectacle lenses and orthokeratology contact lenses^14,17^. It is possible that the degree of myopic defocus is different between the two lenses. Overall, the whole myopic defocus volume is assumed to be higher in the HAL lens than in the DIMS lens, which leads to slower myopia progression and axial growth using HAL lenses.

In this study, we observed that the unadjusted, standardized, 1-year mean myopia progression for HAL lens wearers was − 0.34 D with an AL increase of 0.17 mm. The outcomes are slightly higher than those of the previous 1-year 3-arm randomized control trial (RCT) comparing HAL, slightly aspherical lenslets (SAL), and single-vision lenses (− 0.27 D SER change and 0.13 mm AL increase in the HAL group)^14^. The raw mean SER and AL changes were − 0.63 D and 0.28 mm for the children wearing DIMS lenses in the present study. In contrast, a previous RCT comparing DIMS lenses and single-vision lenses showed less myopia progression as well as AL increase at the 1-year follow-up for DIMS users (− 0.17 D, 0.11 mm)^12^. Table 3 summarises the characteristics and outcomes of the present and previous studies.

**Table 3:** Characteristics and unadjusted 1-year outcomes of the subjects in different studies.

Spectacle lens type	Study	Study initiation date	Region	Age (years)	Baseline SER (D)	SER change (D)	Baseline AL (mm)	AL change (mm)
				Mean (SE)				
HAL	The present study	September 2020	Mainland China	9.57 (0.16)	− 2.24 (0.10)	− 0.34 (0.04)*	24.35 (0.09)	0.17 (0.02)*
	Bao et al.^14^	July 2018	Mainland China	10.70 (0.20)	− 2.70 (0.14)	− 0.27 (0.06)	24.76 (0.09)	0.13 (0.02)
DIMS	The present study	September 2020	Mainland China	10.29 (0.26)	− 2.45 (0.19)	− 0.63 (0.08)*	24.54 (0.13)	0.27 (0.04)*
	Lam et al.^12^	August 2014	Hong Kong, China	10.20 (0.17)	− 2.97 (0.11)	− 0.17 (0.05)	24.70 (0.09)	0.11 (0.02)

*AL* axial length, *D* diopters, *DIMS* defocus incorporated multiple segments, *HAL* highly aspherical lenslets, *SE* standard error, *SER* spherical equivalent refraction.

*Standardized 1-year change.

We assumed that the study period and region are the two main factors that caused the notable discrepancy in the effect for DIMS lenses between the study of Lam et al. and ours. Between 2020 and 2021, because of the COVID-19 pandemic, web-based education was promoted and even required in affected regions, including China^18^. Several studies have shown a myopia surge during this period^19–21^. The DIMS study by Lam et al. was conducted from 2014 to 2017, which was before the COVID-19 pandemic, while the HAL studies of Bao et al. and the present study were conducted mainly during the pandemic. In addition, both the HAL RCT and the present study observed children in mainland China, which has numerous differences in its education system, living environment, and lifestyle compared with those of Hong Kong, China.

In this study, older children had slower axial elongations but showed similar SER changes compared to younger children. It is possible that the process of emmetropization contributed to a significant AL increase without a comparable degree of SER change in young children^22^. In this study, neither sex nor baseline myopic indexes (SER and AL) were associated with myopia progression.

## Strengths of the study

To the best of our knowledge, this was the first study comparing HAL and DIMS lenses, which are two of the most compelling spectacle lenses for myopia control compared with single-vision spectacle lenses^6,11,14,23^. We compared these two lenses in the same population and found that HAL-designed lenses had a more substantial effect than DIMS lenses for the prevention of myopia progression as well as AL elongation in Chinese children.

DIMS lenses had been widely used before HAL lenses were introduced to the market. To control the lockdown effect on myopia progression, we chose the starting point when the first qualified HAL lens user was available and obtained a similar baseline date distribution between the two study lenses^19,20^. Additionally, we found that for the HAL lenses, the average annual change in SER was approximately − 0.34 D, with an increase in AL of 0.17 mm from the real-world data.

## Limitations of the study

There are some limitations in this study. Since this is a retrospective study, some confounders could not be identified, such as time spent outdoors, near-work time, myopia in parents, and duration of wearing spectacle lenses. The average follow-up was approximately nine months, and we used a standardized method to predict 1-year outcomes, which might be slightly different from the actual results for 1 year^24^. However, since the follow-up duration was similar between the two groups, this method should not affect the comparisons between the two types of lenses. Some children did not have cycloplegic refraction in the follow-up tests, and the results might overestimate the progression of myopia. However, we found a corresponding axial elongation associated with SER progression in children^25^. In addition, the data were from Chinese children in only one region. Thus, the results may not be applied to other ethnic children or other areas. Although the study had some limitations, we found a significant difference in myopia control between the two most effective spectacle lenses. Future studies with randomized designs or with locations in other regions are encouraged.

## Conclusion

Spectacle lenses with highly aspherical lenslets (HAL) were more effective in delaying myopia progression and axial elongation than defocus incorporated multiple segments (DIMS) lenses in Chinese children.

## Data Availability

The data that support the findings in this study are available from the corresponding authors on reasonable request.
